# Supplementary material for: The Systems Biology Research Tool: evolvable open-source software
Source: BMC Syst Biol. 2008 Jun 29;2:55. doi: 10.1186/1752-0509-2-55 (PMC2446383; doi:10.1186/1752-0509-2-55)
Supplement: Additional file 1 — SBRT Archive. An archive of the current version of the Systems Biology Research Tool. [file 1752-0509-2-55-S1.zip › sbrt-1.4.0/doc/developers_guide/api/sbrt/kernel/util/class-use/BooleanRelation.html]

Uses of Class sbrt.kernel.util.BooleanRelation


|  |  |  |
| --- | --- | --- |
| **Overview** \| **Package** \| **Class** \| **Use** \| **Tree** \| **Deprecated** \| **Index** \| **Help** | |  |
| PREV   NEXT | **FRAMES**    **NO FRAMES**     **All Classes** |  |


---


## **Uses of Class sbrt.kernel.util.BooleanRelation**

| Packages that use BooleanRelation | |
| --- | --- |
| **sbrt.kernel.fba** | Provides classes and interfaces for creating objects relevant to Flux Balance Analysis. |
| **sbrt.kernel.util** | Provides classes and interfaces for performing utility functions. |

Uses of BooleanRelation in sbrt.kernel.fba

| Methods in sbrt.kernel.fba that return BooleanRelation | |
| --- | --- |
| `BooleanRelation` | `CatalyzedFluxome.getCatalystRule(java.lang.String rxnName)`             Returns the boolean logic rule for catalysts of the reaction with the provided name. |

| Methods in sbrt.kernel.fba that return types with arguments of type BooleanRelation | |
| --- | --- |
| `java.util.Map<java.lang.String,? extends BooleanRelation>` | `CatalyzedFluxome.getCatalystRules()`             Returns the boolean logic rule for the catalysts of all reactions in this fluxome. |

Uses of BooleanRelation in sbrt.kernel.util

| Subclasses of BooleanRelation in sbrt.kernel.util | |
| --- | --- |
| `class` | `And<E>` |
| `class` | `Or<E>` |

---


|  |  |  |
| --- | --- | --- |
| **Overview** \| **Package** \| **Class** \| **Use** \| **Tree** \| **Deprecated** \| **Index** \| **Help** | |  |
| PREV   NEXT | **FRAMES**    **NO FRAMES**     **All Classes** |  |


---
